# Hydrogen sulfide protects against spinal cord pyroptosis via persulfidation of Rac1 after lumbosacral plexus nerve injury

**DOI:** 10.1038/s41420-025-02736-x

**Published:** 2025-10-06

**Authors:** Jianyu Mao, Jiajia Lu, Sheng Wang, Xin Jiang, Jing Li, Qiang Fu, Nan Lu, Lei Zhu, Aimin Chen, Jun Ma

**Affiliations:** 1 Department of Orthopedic Trauma, Shanghai Fourth People’s Hosptial, School of Medicine, TongJi University, Shanghai, China; 2 Department of Orthepaedic, Naval Medical Center of PLA, Naval Military Medical University, Shanghai, China; 3 Department of Orthopaedic Trauma Surgery, Shanghai Changzheng Hospital, Navy Military Medical University, Shanghai, China

**Keywords:** Trauma, Cell death

## Abstract

Pyroptosis, a form of lytic and inflammatory programmed cell death mediated by gasdermin proteins, contributes to progressive spinal cord neurodegeneration following neural trauma. Nevertheless, the regulatory mechanisms governing this process remain inadequately characterized. In this investigation, hydrogen sulfide (H₂S) was identified as an endogenous inhibitor of neuronal pyroptosis, functioning through Rac1-dependent NLRP3 inflammasome signaling. In a rat model of lumbosacral plexus nerve injury, H₂S treatment significantly decreased pyroptosis-associated markers (NLRP3, caspase-1, GSDMD) and enhanced neuronal survival. In vitro, the administration of H₂S effectively mitigated hypoxia-induced neuronal pyroptosis by inhibiting the assembly of the NLRP3 inflammasome. Mechanistically, our findings indicate that H₂S selectively persulfidates Rac1, inhibiting its GTPase activity and reducing reactive oxygen species (ROS) production, both of which are critical for inflammasome priming. Molecular dynamics simulations and site-directed mutagenesis further confirmed that H₂S persulfidation of Rac1 at Cys178 alters its conformation, thereby suppressing NLRP3 inflammasome activation. Taken together, our findings reveal a novel post-translational regulatory mechanism by which H₂S mitigates pyroptotic neuronal death through Rac1 persulfidation, highlighting the H₂S-Rac1 axis as a promising therapeutic target for neuroprotection in pyroptosis-related neurodegeneration.

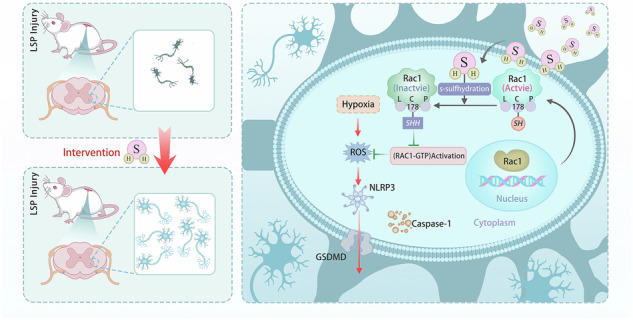

## Introduction

Lumbosacral plexus nerve injury (LSPI), often resulting from high-energy trauma such as severe pelvic fractures, leads to irreversible motor-sensory deficits in the lower limbs and progressive neurodegeneration of the spinal cord [1, 2]. Following peripheral nerve injury, the retrograde degeneration of spinal neurons involves complex pathophysiological processes, including excitotoxicity, oxidative stress, and chronic neuroinflammation, which collectively contribute to irreversible synaptic loss and axonal disintegration [3–5]. Despite advancements in microsurgical techniques and therapies based on neurotrophic factors, persistent secondary neuronal loss driven by inflammatory signaling remains a significant barrier to functional recovery [6–8]. Emerging evidence highlights that pyroptosis-a lytic form of programmed cell death initiated by inflammasome activation and GSDMD dependent cytomembrane permeabilization is a critical driver of post-traumatic neurodegeneration [9–11]. In contrast to apoptosis, pyroptosis facilitates the release of proinflammatory cytokines, such as IL-1β and IL-18, via GSDMD pores, consequently intensifying neurotoxic immune responses and sustaining a cycle of neuronal damage [12, 13]. However, the spatiotemporal regulation of pyroptotic pathways in the spinal cord post-LSPI, especially their interaction with redox-sensitive signaling cascades, remains inadequately characterized.

Hydrogen sulfide (H₂S), recognized as a vital gasotransmitter, has emerged as a significant neuroprotective agent due to its ability to modulate oxidative stress, mitochondrial function, and inflammatory signaling through diverse mechanisms [14, 15]. Endogenously synthesized via several key enzymes, including cystathionine β-synthase (CBS), cystathionine γ-lyase (CSE), and 3-mercaptopyruvate sulfurtransferase (3-MST) [16], H₂S exhibits direct antioxidant properties. Furthermore, H₂S promotes the persulfidation of cysteine residues, a redox-sensitive post-translational modification that dynamically regulates protein function [17]. For example, the persulfidation of Keap1 disrupts its interaction with Nrf2, thereby augmenting the expression of genes governed by the antioxidant response element (ARE), while modifications to NF-κB subunits inhibit pro-inflammatory transcription [18, 19]. In spinal cord injury models, H₂S donors, including sodium hydrosulfide (NaHS), have shown effectiveness in mitigating gliosis and preventing the disruption of the blood-spinal cord barrier. Nevertheless, their potential involvement in the modulation of pyroptosis, especially in relation to degenerative changes induced by spinal cord injury, has yet to be investigated. Importantly, the diverse effects of H₂S are influenced by factors such as concentration, pharmacokinetics, and subcellular localization, highlighting the necessity for further research to elucidate its therapeutic mechanisms in spinal cord neurodegeneration.

Rac1, a small GTPase, is pivotal in regulating cytoskeletal remodeling, the assembly of NADPH oxidase (NOX), and the activation of redox-sensitive transcription factors [20]. Upon activation in its GTP-bound state, Rac1 enhances NOX2-dependent superoxide production, which subsequently triggers the activation of the NLRP3 inflammasome through increased mitochondrial reactive oxygen species (ROS) and the oxidation of thioredoxin-interacting protein (TXNIP) [21, 22]. Moreover, the Rac1/ROS-mediated activation of the NLRP3 inflammasome exacerbates neuroinflammation in post-traumatic spinal cord injury, initiating a positive feedback loop that amplifies pyroptotic signaling cascades [23]. Importantly, Rac1 possesses a redox-sensitive cysteine-rich domain that undergoes post-translational modifications, such as S-nitrosylation and persulfidation, which stabilize Rac1 in its inactive GDP-bound conformation [24]. Therefore, we hypothesize that H₂S may confer neuroprotection by promoting the persulfidation of Rac1, thereby attenuating NLRP3-mediated pyroptotic signaling in spinal cord neurons following LPSI.

In this study, we aimed to investigate the role of pyroptosis in the spinal cord after LPSI, explore the potential protective effect of H₂S against neuronal pyroptosis, and elucidate the underlying mechanism through which H₂S exerts its anti-pyroptotic effect, with a specific focus on the involvement of Rac1. This research may offer a novel therapeutic approach for the management of sacral plexus injuries.

## Methodes

### Animal experiments

Adult male Sprague-Dawley (SD) rats, with a body weight range of 200–250 g, were employed in this study. Rats were assigned to experimental groups with randomization. A unilateral nerve injury model was precisely established under general anesthesia using isoflurane. Specifically, the L4–L6 nerve roots were surgically transected to induce a lumbosacral plexus nerve injury. In the sham-operated cohort, rats underwent identical surgical procedures without the severance of nerve roots. Post-operative assessments of Basso–Beattie–Bresnahan (BBB) locomotor scores and stride length were conducted by two experienced observers who were blinded to the experimental groups, at one day post-injury. For the therapeutic intervention, GYY4137(Sigma, MO, USA) was administered to the rats at a dosage of 50 mg/kg body weight, whereas control animals received an equivalent volume of a 0.4% DMSO vehicle solution (Aladdin, Shanghai, China). The sample size of 3 rats per group was determined to balance practical feasibility with the ability to detect the pre-specified effect size, considering the study design and expected variability. All animal experiments were conducted in accordance with the Guide for the Care and Use of Laboratory Animals and approved by the Animal Ethics Committee of the Navy Military Medical University (Approval No.: 2025H005).

### Histological analysis

The rats were euthanized, and the spinal cord segments corresponding to the site of injury were meticulously dissected. For hematoxylin and eosin (HE) staining, the tissues were fixed in 4% paraformaldehyde(Aladdin, Shanghai, China), embedded in paraffin, sectioned at a thickness of 5 μm, and subsequently stained. Microscopic examination was conducted to identify histopathological alterations, such as neuronal pallor, cell shrinkage, and widening of the glial intercellular spaces. For immunofluorescence co-staining, the sections were blocked with 5% bovine serum albumin (Sigma, MO, USA) and incubated overnight at 4 °C with primary antibodies targeting the pyroptosis executor GSDMD(Abcam, Cambridge, UK) and the neuronal marker NeuN(Abcam, Cambridge, UK). Following washing, appropriate fluorescently labeled secondary antibodies were applied, and the nuclei were counterstained with DAPI. Fluorescence images were acquired using a confocal microscope to evaluate the expression and co-localization of GSDMD and NeuN in spinal cord neurons at various post-injury time points.

### H_2_S measurement

Total RNA was extracted from spinal cord tissues using TRIzol reagent (Invitrogen, CA, USA). Reverse transcription was performed to synthesize cDNA, and qRT-PCR was carried out using specific primers for endogenous H₂S synthase genes, namely 3-mercaptopyruvate sulfurtransferase (3MST), cystathionine-γ-lyase (CSE), and cystathionine-β-synthase (CBS). The expression levels of these genes were normalized to the housekeeping gene GAPDH, and relative fold changes were calculated using the 2⁻ΔΔCt method to evaluate the change of H₂S related gene expression after injury.

### Cell culture

Primary spinal cord neurons were isolated from neonatal SD rats. The spinal cords were carefully dissected and digested with 0.25% trypsin (Sigma, MO, USA) for 15 min at 37 °C. After trituration, the cell suspension was passed through a 70 μm cell strainer to obtain single cell suspensions. Neurons were seeded onto poly-D-lysine (Aladdin, Shanghai, China) coated 24 well plates or glass coverslips in Neurobasal medium supplemented with B27, GlutaMAX, and penicillin streptomycin. After 24 h incubation, the medium was replaced with fresh medium. To establish a pyroptosis model, neurons were exposed to hypoxia (1% O₂, 5% CO₂, and 94% N₂), while the normoxic control group was maintained in a standard incubator (5% CO₂, 95% air). The morphological changes of neurons were observed under an inverted phase contrast microscope (Nikon, Tokyo, Japan) at different time points.

### Transcriptome sequencing

To delineate H₂S mediated transcriptional regulation in hypoxia-induced pyroptosis, RNA sequencing was conducted. Total RNA was isolated using the RNeasy Mini Kit (Qiagen, Hilden, Germany), with RNA integrity verified by Agilent 2100 Bioanalyzer (RIN > 9.0). Strand-specific libraries were prepared using the NEBNext® Ultra™ II Directional RNA Library Prep Kit, followed by 150 bp paired-end sequencing on an Illumina NovaSeq 6000 platform. Raw reads were quality-trimmed using Trimmomatic, aligned to the Rattus norvegicus genome Rn6 via STAR, and quantified using featureCounts. Differential expression analysis was performed with DESeq2, applying an FDR-adjusted *p* < 0.05 and |log2(fold change)| >1 as significance thresholds. Gene ontology (GO) and KEGG pathway enrichment analyses were conducted using clusterProfiler.

### Cell transfection

Lentiviral vectors carrying short-hairpin RNA targeting Rac1 or nontargeting control shRNA were purchased (Santa Cruz Biotechnology, Dallas, USA). Neuronal cells were transfected with lentiviral particles according to the manufacturer’s instructions. Briefly, cells at 60–70% confluence were incubated with lentiviral vectors in the presence of polybrene (8 μg/mL) for 12 h. The medium was then replaced with fresh medium, and cells were cultured for an additional 48 h. Western blot was performed to confirm the successful knockdown of Rac1 protein expression. Whole cell lysates were prepared, and protein concentration was determined using a BCA protein assay kit (Thermo Fisher Scientific, MA, USA). Equal amounts of protein were separated by SDS-PAGE, transferred to PVDF membranes, blocked with 5% non fat milk, and incubated with primary antibody against Rac1(Abcam, Cambridge, UK) overnight at 4 °C. After washing, appropriate horseradish peroxidase conjugated secondary antibody was applied, and protein bands were visualized using an enhanced chemiluminescence detection system.

### Cell immunofluorescence staining

Neuronal cells grown on glass coverslips were fixed with 4% paraformaldehyde for 15 min, permeabilized with 0.1% Triton X-100 (Aladdin, Shanghai, China) for 10 min, and blocked with 5% bovine serum albumin (Aladdin, Shanghai, China) for 1 h. Then, they were incubated with primary antibodies against NLRP3, GSDMD, and NeuN overnight at 4 °C. After washing with PBS, fluorescently-labeled secondary antibodies were applied for 1 h at room temperature. Nuclei were counterstained with DAPI for 5 min. Coverslips were mounted onto glass slides using anti-fade mounting medium, and fluorescence images were captured using a confocal microscope. The intensity of fluorescence signals for each protein was quantified using ImageJ software to assess the occurrence of pyroptosis and the effect of different treatments, such as H₂S donor treatment or gene silencing.

### Measurement of ROS production

Neuronal cells were loaded with 10 μM of the ROS sensitive fluorescent probe DCFH-DA (Sigma, MO, USA) for 30 min at 37 °C in the dark. After washing with PBS, cells were subjected to hypoxia or treated with H₂S donor GYY4137 in the presence or absence of DTT (a sulfhydration inhibitor). Fluorescence intensity was measured using a fluorescence microplate reader at an excitation wavelength of 488 nm and an emission wavelength of 525 nm. The relative ROS production was calculated by normalizing the fluorescence intensity of treated cells to that of untreated control cells.

### Rac1 GTPase activity assay

Active Rac1 (GTP-bound) levels were quantified using the G-LISA® Activation Assay Kit (Cytoskeleton, CO, USA) following manufacturer guidelines. Primary spinal cord neurons were lysed in ice-cold buffer, and protein normalized lysates were incubated in Rac1-GTP affinity plates. After sequential incubation with anti-Rac1 primary and HRP-conjugated secondary antibodies, luminescence signals were measured using a GloMax® Discover Microplate Reader.

### NADPH/NADP+ ratio assay

NADPH and NADP+ levels were quantified using a NADPH/NADP+ assay kit (Beyotime Biotechnology, Shanghai, China) according to the manufacturer’s protocols. Briefly, 1 × 10⁶ cells were harvested and lysed via three cycles of freezing at −80 °C for 10 min and thawing on ice. The lysate was split into two aliquots: one was heated at 60 °C to deplete NADP⁺ (retaining NADPH), and the other was kept on ice (containing both NADP⁺ and NADPH). In the working buffer, NADP⁺ in the unheated aliquot was reduced to NADPH, and total NADPH (endogenous + generated) facilitated WST-8 reduction to formazan. Absorbance of the orange formazan was measured at 450 nm. NADPH level was derived from the heated aliquot, NADP⁺ level from the difference between unheated and heated aliquots, and their ratio was calculated accordingly.

### Sulfhydration assay

Purified Rac1 protein was incubated with an GYY4137 at 37 °C for 2 h. The reaction mixture was then subjected to biotin-switch assay to detect sulfhydration. Briefly, the protein was first incubated with methyl-methanethiosulfonate to block free thiols, followed by incubation with biotin-HPDP (Thermo Fisher Scientific, MA, USA) in the presence of ascorbic acid to label the sulfhydrated cysteine residues. The biotin labeled proteins were then captured using streptavidin-agarose beads, and the sulfhydrated Rac1 protein was detected by Western blot using an antibody against Rac1. In parallel, neuronal cells were treated with H₂S donor or H₂S donor plus DTT (Sigma, MO, USA), and cell lysates were analyzed for the sulfhydration of endogenous Rac1 protein using the same biotin-switch assay.

### Molecular dynamics simulation of sulfhydrylation

A 50-ns kinetic simulation was conducted on Rac1 protein with GYY4137 using the GROMACS software package. The initial structure of Rac1 was obtained from the Protein Data Bank. The GYY4137 molecule was docked onto Rac1 using AutoDock Vina, and the docked complex was solvated in a cubic box of TIP3P water molecules. Counterions were added to neutralize the system. The simulation was carried out under periodic boundary conditions with a constant temperature of 310 K and a constant pressure of 1 bar. The root mean square deviation (RMSD) and solvent-accessible surface area of Rac1 were calculated during the simulation. The conformational changes of Rac1 were analyzed by visual inspection of the trajectory files using VMD software to understand the impact of H₂S-induced sulfhydrylation on Rac1 activity.

### Site directed mutagenesis of Rac1

To identify the specific cysteine residue responsible for H₂S mediated Rac1 sulfhydrylation, site-directed mutagenesis was performed on the Rac1 gene. The Rac1 cDNA was cloned into a pcDNA3.1 vector. Using the QuikChange Lightning Multi Site-Directed Mutagenesis Kit (Agilent Technologies, CA, USA), the cysteine at position 178 (Cys178) was substituted with serine (C178S) via overlapping PCR with mutagenic primers. Mutant constructs were confirmed by Sanger sequencing. Neuronal cells were transfected with wild-type (WT) or C178S mutant Rac1 plasmids using Lipofectamine 3000 (Invitrogen, CA, USA) according to the manufacturer’s protocol.

### Quantification of mRNA and qPCR

Total RNA was extracted from spinal cord tissues or neuronal cells using TRIzol reagent. RNA concentration and purity were determined by spectrophotometry. Reverse transcription was performed using a high capacity cDNA reverse transcription kit (Applied Biosystems, CA, USA). qRT-PCR was carried out using SYBR Green Master Mix on a real time PCR system. Specific primers were designed for pyroptosis associated genes (NLRP3, GSDMD, Caspase-1) and housekeeping gene GAPDH. The reaction conditions included an initial denaturation at 95 °C for 10 min, followed by 40 cycles of 95 °C for 15 s and 60 °C for 1 min. The relative gene expression levels were calculated using the 2⁻ΔΔCt method, with β-actin as the internal control. The primers used for expression analysis were listed in Table 1

**Table 1:** Premiers for qRT-PCR analysis.

**Oligonucleotides**		
Primesr: NLRP3(RT-PCR) F- GAAACTCTGGTTGGTCAGC R- AGAGAATGGTTGGAGCTCAG	IDT	NM_001191642.1
Primesr: Caspase1(RT-PCR) F- CGACTCTGGAGAACTGGTG R- TGGGTTTCACTCAACCCAG	IDT	NM_009807.2
Primesr: 3MST(RT-PCR) F- GCTCAGTAAACATCCCATTCA R- GCCATCGTAGACAGGCACAT	IDT	NM_138843.2
Primesr: CSE(RT-PCR) F- GTTGTCATGGGCTTAGTG R- CTCGGCAGCAGAGGTA	IDT	NM_017074.2
Primesr: CBS(RT-PCR) F- GCTCAGTAACACTCCCATTCA R- GCCATCGTAGACAGGCACAT	IDT	NM_012522.2
Primesr: GSDMD(RT-PCR) F- ATTGGCTCTGAATGGGATA R- GTACGGCAAGCAGACTAAA	IDT	NM_001400993.1
Primesr: Rac1 (RT-PCR) F- CCTGCTCATCAGTTACACGACCA R- GTCCCAGAGGCCCAGATTCA	IDT	NM_134366.1
Primesr: β-actin(RT-PCR) F- GGCTGTATTCCCCTCCATCG R-CCAGTTGGTAACAATGCCATGT	IDT	NM_031144.3

### Western blot analysis

Cells or tissues were lysed in RIPA buffer containing protease and phosphatase inhibitors. Protein concentration was determined using a BCA protein assay kit (Pierce, IL, USA). Equal amounts of protein were separated by SDS-PAGE on 10–12% polyacrylamide gels and transferred to PVDF membranes. Membranes were blocked with 5% nonfat milk in Tris-buffered saline with Tween 20 (TBST) for 1 h at room temperature and then incubated with primary antibodies against NLRP3, Caspase-1, GSDMD,NeuN, and Rac1 overnight at 4 °C. After washing with TBST, appropriate horseradish peroxidase-conjugated secondary antibodies were applied for 1 h at room temperature. Protein bands were visualized using an enhanced chemiluminescence detection system, and the intensity of bands was quantified using ImageJ software to analyze the changes in protein expression levels under different experimental conditions.

### Statistical analysis

Data were collected from three or more independent experiments. GraphPad Prism software (Version 5.01, GraphPad Software, La Jolla, CA) was used to analyze the data. Data was reported as mean ± standard deviation (SD). Statistical analyses were performed by independent *t*-test for two groups comparison, or one-way analysis of variance followed by Tukey’s test for more than three groups comparison. Statistical significance was set at *p < 0.05 and **p < 0.01.

## Results

### Pyroptosis accumulates in the spinal cord following lumbosacral plexus nerve injury

**Fig. 1:**
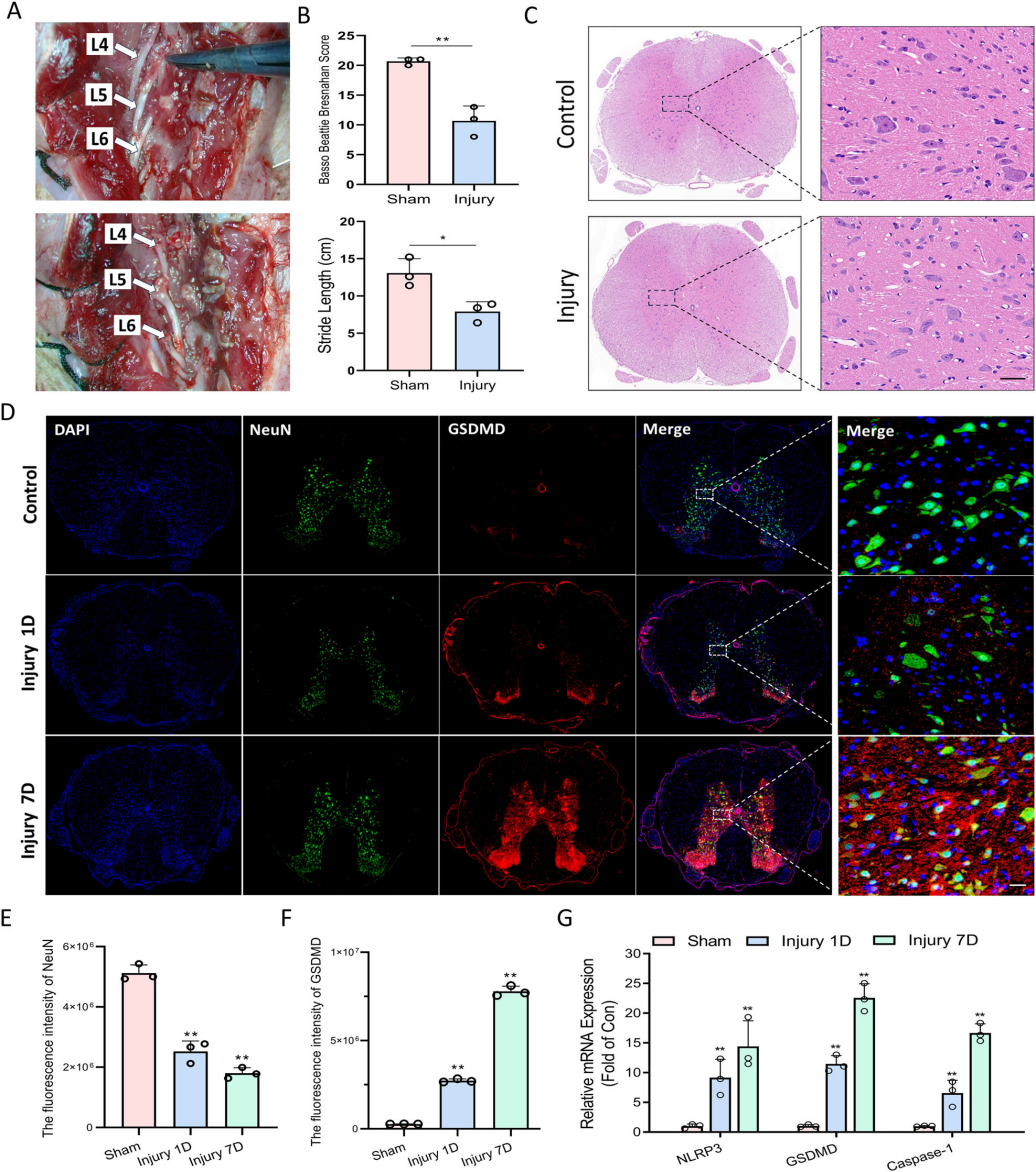
Pyroptosis accumulates in the spinal cord following sacral plexus injury. **A** Surgical images depicting the establishment of the sacral plexus injury model by severing the L4-L6 nerve roots in SD rats. **B** Basso–Beattie–Bresnahan (BBB) scores and stride length, demonstrating significant functional impairment in the injury group. *n* = 3, *P < 0.05, **P < 0.01. **C** HE staining of spinal cord tissues, Scale bar, 20 μm. **D**–**F** Immunofluorescence co-staining for Gasdermin D (GSDMD, red) and Neuronal Nuclei (NeuN, green), Scale bar, 20 μm. Progressive upregulation of GSDMD and downregulation of NeuN were observed in the injured spinal cords post-injury. *n* = 3, *P < 0.05, **P < 0.01. **G** qRT-PCR analysis showing a time-dependent elevation of pyroptosis-associated mRNA in the injured spinal cords. *n* = 3, *P < 0.05, **P < 0.01.

To examine the dynamics of pyroptosis following lumbosacral plexus nerve injury, a unilateral nerve injury model was developed in Sprague-Dawley rats through the surgical transection of the L4-L6 nerve roots (Fig. 1A). Postoperative assessments, including Basso–Beattie–Bresnahan (BBB) locomotor scores and stride length measurements, demonstrated significant functional deficits in the injury group relative to the sham controls (Fig. 1B), thereby confirming the successful establishment of the model. Histopathological evaluation using hematoxylin and HE staining revealed neuronal pallor and expanded glial intercellular spaces in the injured spinal cords (Fig. 1C). Immunofluorescence co-staining for the pyroptosis executor GSDMD and the neuronal marker NeuN indicated a progressive upregulation of GSDMD and a concomitant downregulation of NeuN post-injury (Fig. 1D–F). Correspondingly, quantitative qRT-PCR analysis demonstrated a time-dependent increase in the expression levels of pyroptosis-related transcripts, including NLRP3, GSDMD, and Caspase-1, in the injured spinal cords (Fig. 1G). Collectively, these findings substantiate that lumbosacral plexus nerve injury precipitates progressive pyroptosis in spinal cord neurons.

### Hypoxia triggers pyroptosis in primary spinal cord neurons

**Fig. 2:**
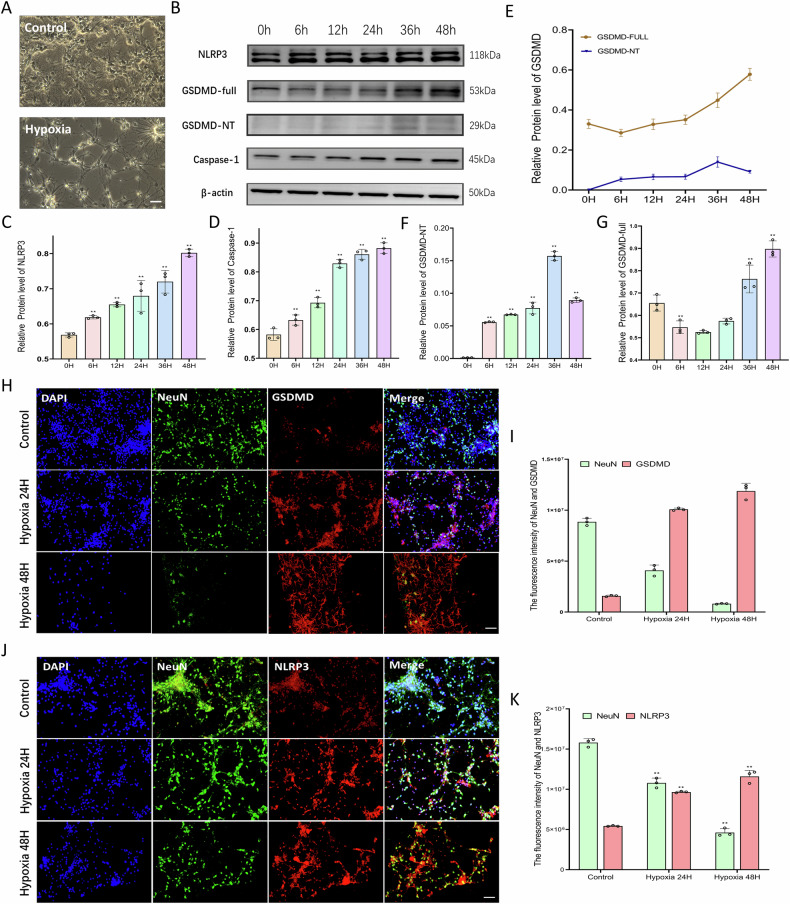
Hypoxia triggers pyroptosis in primary spinal cord neurons. **A** Morphological observations of primary spinal cord neurons in each group, Scale bar, 50 μm. **B**–**G** Western blot analysis of pyroptosis-related proteins (NLRP3, GSDMD, GSDMD-NT, Caspase-1) in neurons under hypoxia. *n* = 3, *P < 0.05, **P < 0.01. **H**, **I** Immunofluorescence staining and Quantitative analysis for NeuN (green), GSDMD (red), Hypoxia induces GSDMD upregulation and NeuN downregulation. Scale bar, 50 μm. *n* = 3, *P < 0.05, **P < 0.01. **J**, **K** Immunofluorescence staining and Quantitative analysis for NeuN (green), NLRP3 (red), Hypoxia induces NLRP3 upregulation and NeuN downregulation. Scale bar, 50 μm. *n* = 3, *P < 0.05, **P < 0.01.

Subsequently, primary spinal cord neurons were isolated and cultured from Sprague-Dawley rats to investigate the characteristics of neuronal pyroptosis. Consistent with previous studies [25], a hypoxic intervention was employed to establish a model of pyroptosis. Neurons subjected to hypoxia displayed shrunken somata and retracted processes, in contrast to the control group, which maintained intact morphology (Fig. 2A). Western blot analyses were conducted to assess changes in the expression of proteins associated with neuronal pyroptosis following hypoxic exposure (Fig. 2B). The findings revealed that with prolonged hypoxic conditions, the expression levels of NLRP3 and Caspase-1 proteins progressively increased (Fig. 2C, D). Additionally, hypoxia was found to enhance the expression of both full-length GSDMD and its N-terminal fragment (GSDMD-NT) (Fig. 2E–G). Immunofluorescence assays further confirmed the concurrent upregulation of NLRP3 and GSDMD, alongside a reduction in NeuN expression under hypoxic conditions (Fig. 2H–K), thereby demonstrating hypoxia-induced neuronal pyroptosis.

### Exogenous H₂S attenuates neuronal pyroptosis

**Fig. 3:**
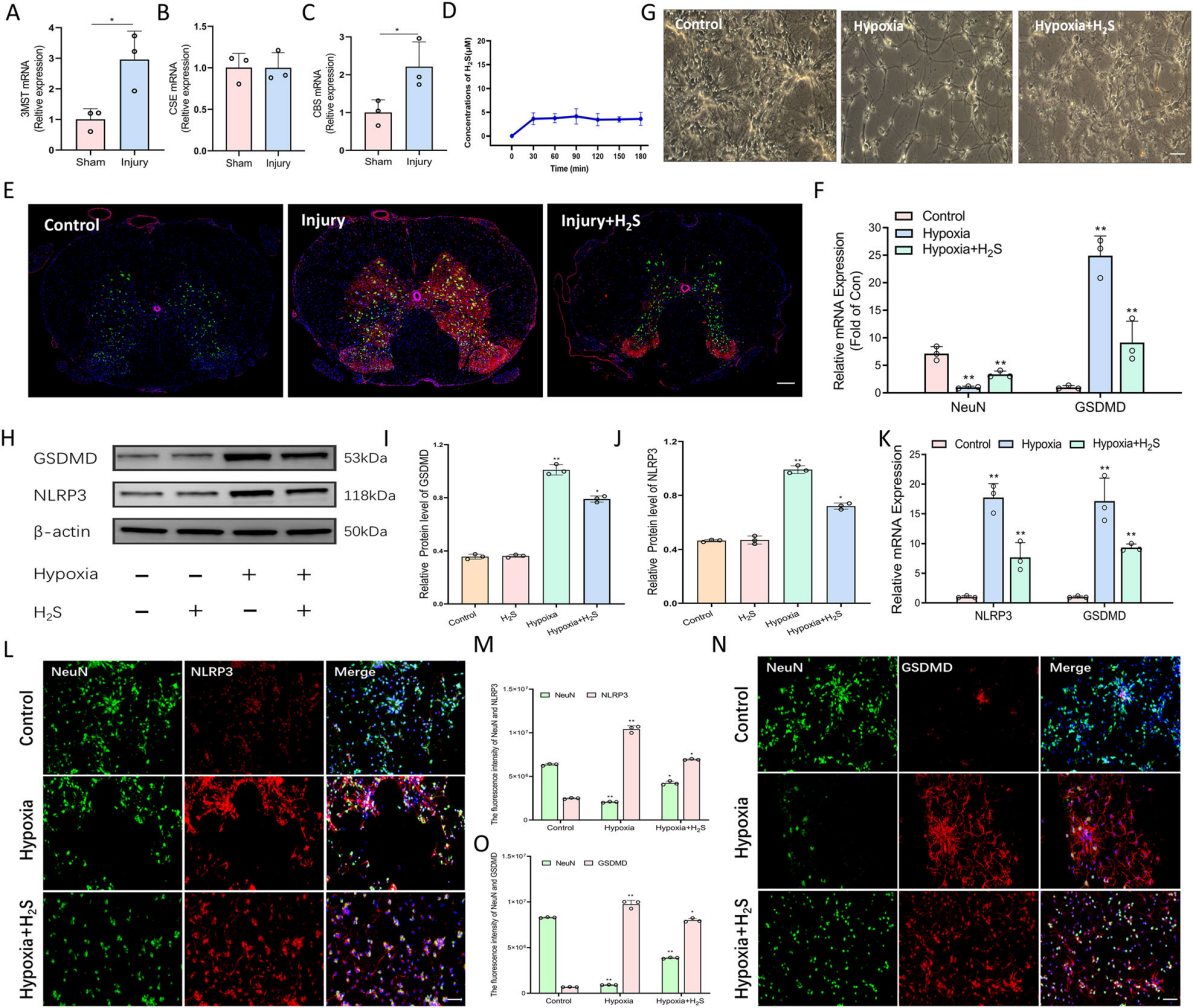
Exogenous H₂S attenuates neuronal pyroptosis. **A**–**C** qRT-PCR analysis of H₂S synthase mRNA levels (3MST, CSE, CBS) in spinal cords. *n* = 3, *P < 0.05, **P < 0.01. **D** Change of H_2_S concentration released by GYY4137, *n* = 3. **E**, **F** H₂S suppressed GSDMD expression and preserved NeuN immunoreactivity in injured spinal cords. Scale bar, 200 μm. *n* = 3, *P < 0.05, **P < 0.01. **G** Morphological observation of primary neurons in each group. Scale bar, 50 μm. **H**–**J** Western blot and quantification revealed H₂S treatment reversed hypoxia-induced upregulation of NLRP3 and GSDMD proteins. *n* = 3, *P < 0.05, **P < 0.01. **K** qRT-PCR showed H₂S reduced hypoxia-driven NLRP3 and GSDMD mRNA elevation. *n* = 3, *P < 0.05, **P < 0.01. **L**, **M** Immunofluorescence staining and quantitative analysis of NeuN (green), NLRP3(red) in each group. Scale bar, 100 μm. *n* = 3, *P < 0.05, **P < 0.01. **N**, **O** Immunofluorescence staining and quantitative analysis of NeuN (green), GSDMD (red) in each group. Scale bar, 50 μm. *n* = 3, *P < 0.05, **P < 0.01.

Given the documented role of H₂S in the regulation of pyroptosis, we investigated the expression of endogenous H₂S synthases following injury. The injured spinal cords demonstrated decreased mRNA levels of 3MST and CSE compared to sham controls, whereas CBS levels remained unchanged (Fig. 3A–C). Notably, GYY4137, the H₂S donor used in this study, was confirmed to slowly release H₂S in vitro (Fig. 3D). Systemic administration of the GYY4137 in injured rats resulted in the suppression of GSDMD expression and preservation of NeuN immunoreactivity (Fig. 3E, F). In vitro experiments revealed that GYY4137 treatment alleviated hypoxia-induced neuronal degeneration (Fig. 3G) and reversed the hypoxia-induced upregulation of NLRP3 and GSDMD at both mRNA and protein levels (Fig. 3H–K). Immunofluorescence analysis further confirmed that GYY4137 mediated the suppression of NLRP3/ASC speck formation and the preservation of NeuN (Fig. 3L–O), suggesting that H₂S mitigates pyroptosis in both in vivo and in vitro models.

### Rac1 mediates the inhibitory effect of H_2_S on spinal cord neuron pyroptosis

**Fig. 4:**
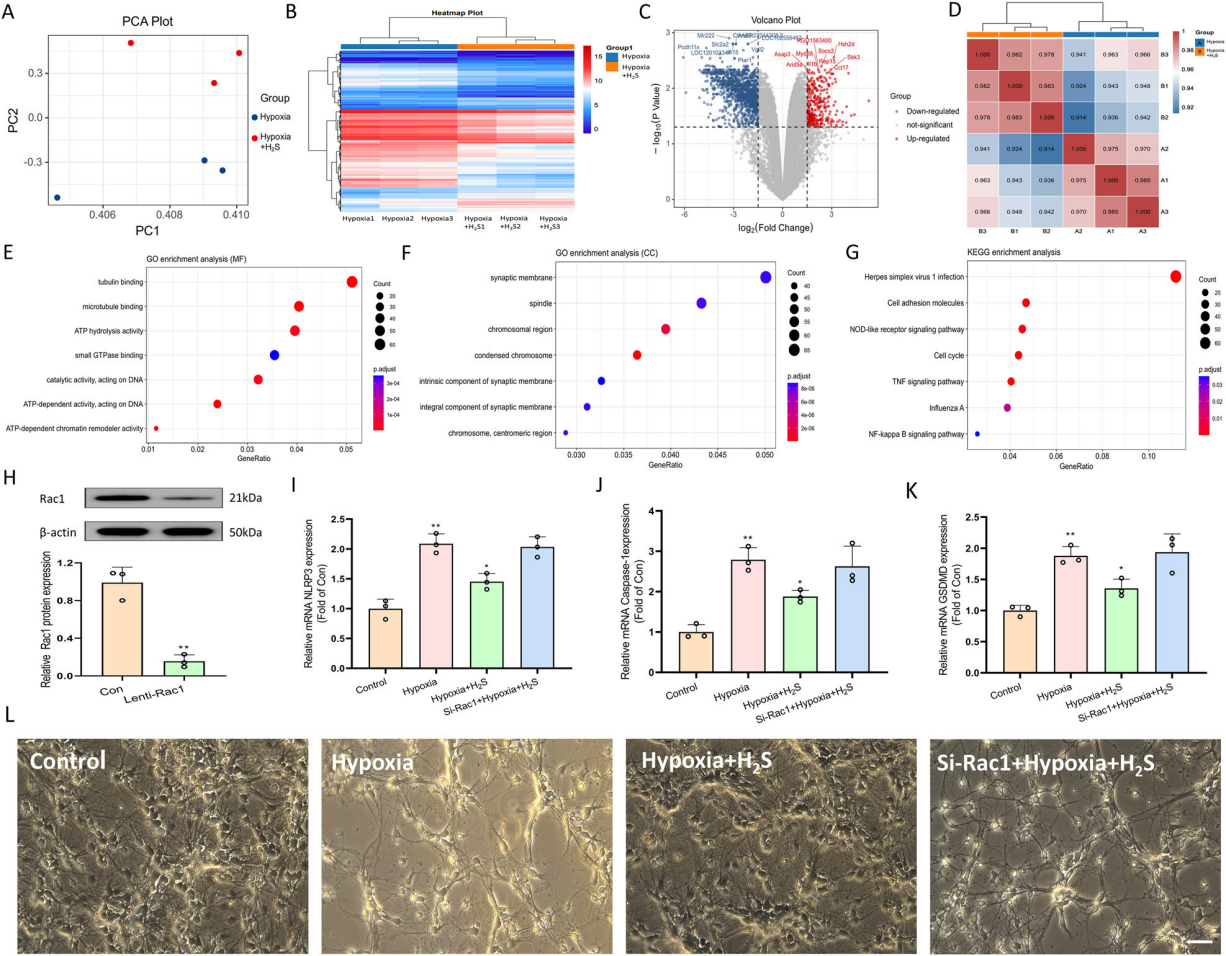
Rac1 mediates the inhibitory effect of H₂S on spinal cord neuron pyroptosis. **A** Principal component analysis (PCA) plot showing significant clustering differences in gene expression between two groups. **B** Heatmap of differentially expressed genes (DEGs). **C** Volcano plot identifying 294 upregulated and 2074 downregulated genes. **D** Sample correlation heatmap clarifying relationships among samples. **E**, **F** GO enrichment analysis showing significant terms. **G** KEGG enrichment analysis highlighting pathways like NF-κB. **H** Western blot confirming Rac1 gene silencing via lentiviral transfection. **I** qRT-PCR results reveal that H₂S suppressed NLRP3, Caspase-1, and GSDMD expression in hypoxia-treated neurons, but this inhibitory effect was reduced when Rac1 was silenced. *n* = 3, *P < 0.05, **P < 0.01. **L** Light microscopy images of each group. Scale bar, 50 μm.

To further investigate the role of H₂S in mitigating spinal cord neuron pyroptosis, transcriptome sequencing was performed on neuronal cells with and without H₂S exposure. PCA revealed significant clustering differences between the hypoxia group and the hypoxia group supplemented with H₂S (Fig. 4A), indicating notable differences in gene expression. A total of 2368 differentially expressed genes were identified, with 294 genes upregulated and 2074 genes downregulated (Fig. 4B, C). A heatmap, color-coded by correlation values ranging from 0.914 to 1.000, illustrated pairwise correlations among the samples, thereby elucidating sample relationships (Fig. 4D). GO and KEGG enrichment analyses revealed significant enrichments, such as small GTPase binding in molecular function, synaptic membrane-associated cellular components, and the NF-κB signaling pathway, suggesting Rac1 as a potential target for the inhibitory effect of H₂S on cell pyroptosis (Fig. 4E–G). Experimentally, lentiviral transfection was employed to silence the Rac1 gene in neuronal cells, with successful silencing confirmed by Western blot analysis (Fig. 4H). This resulted in a marked reduction in the inhibitory effect of H₂S on neuronal pyroptosis, as H₂S was no longer able to downregulate key pyroptosis-related proteins, including NLRP3, Caspase-1, and GSDMD (Fig. 4I–K). Further examination using light microscopy revealed that with the silencing of the Rac1 gene, H₂S lost its capacity to alleviate hypoxia-induced morphological alterations in neurons and preserve their normal characteristics (Fig. 4L). Collectively, these findings indicate that Rac1 may serve as a critical target through which H₂S exerts its inhibitory effects on cell pyroptosis.

### Hydrogen sulfide inhibits Rac1 activity by sulfhydration

**Fig. 5:**
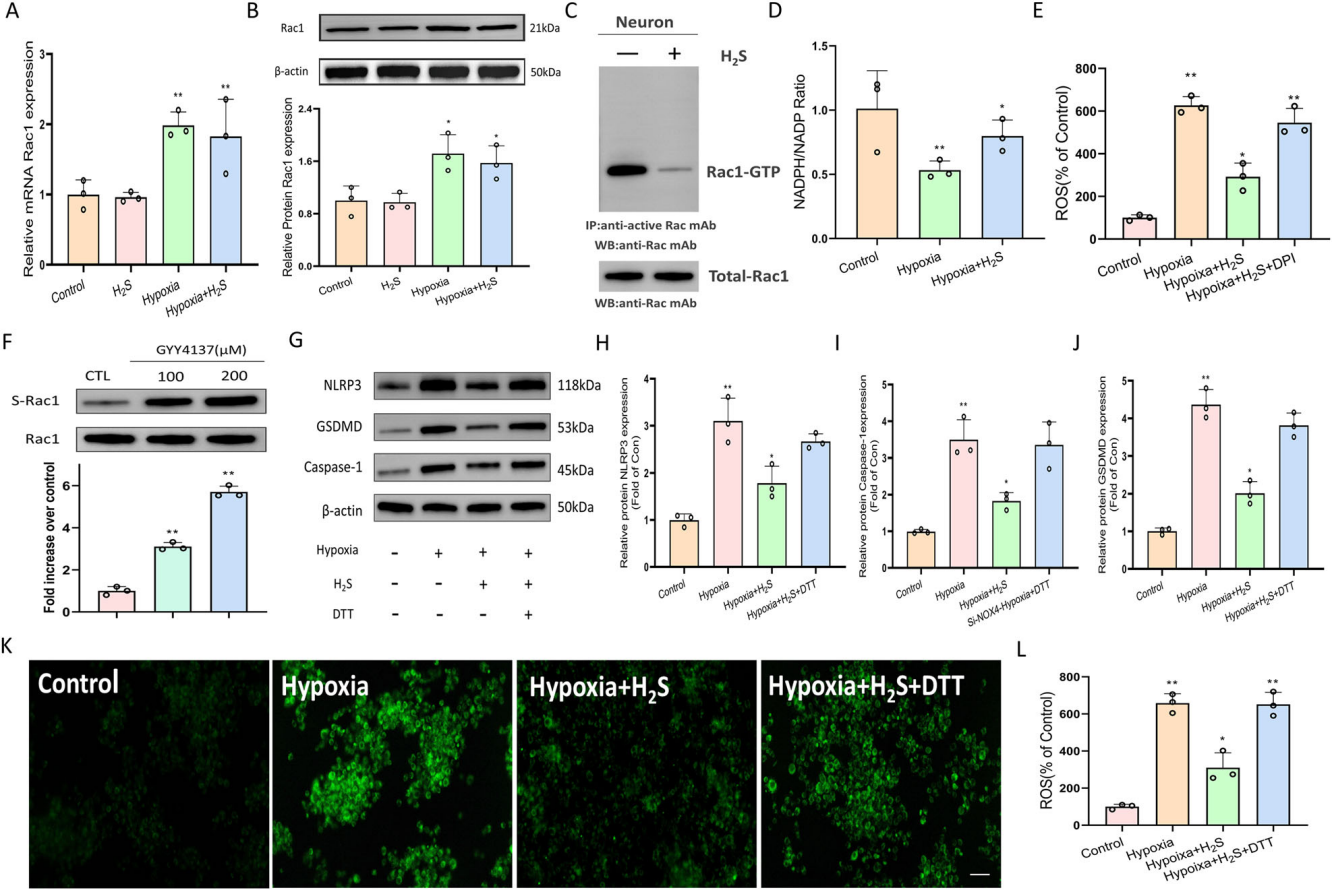
H_2_S inhibits Rac1 activity by sulfhydration. **A**, **B** qRT-PCR and Western blot analyses reveal that H₂S does not regulate the transcriptional expression or protein level of Rac1 in neuronal cells. **C** H₂S intervention inhibits Rac1 activity in neurons, detected via immunoprecipitation and Western blot for Rac1-GTP. **D** The ratio of NADPH to NADP⁺ in neuronal cells was measured across groups. *n* = 3, *P < 0.05, **P < 0.01. **E** ROS levels in neuronal cells were quantified for each group. *n* = 3, *P < 0.05, **P < 0.01. **F** Sulfhydration of purified Rac1 protein. *n* = 3, *P < 0.05, **P < 0.01. **G**–**J** Western blot and quantitative analysis reveal that pretreatment with DTT abolishes the H₂S-induced inhibition of NLRP3, Caspase-1, and GSDMD expression in hypoxia-stimulated neurons. *n* = 3, *P < 0.05, **P < 0.01. **K**, **L** Fluorescence staining and quantification of ROS in each group. Scale bar, 50 μm. *n* = 3, *P < 0.05, **P < 0.01.

Subsequently, we explored the mechanism through which Rac1 mediates the inhibitory effect of H₂S on neuronal pyroptosis. PCR and Western blot analyses revealed that, within a pyroptosis-inducing environment, H₂S did not influence the transcription or expression of Rac1 in neuronal cells (Fig. 5A, B). Notably, H₂S intervention was found to suppress Rac1 activity, specifically by inhibiting its GTP-binding capacity (Fig. 5C). Additionally, NADPH oxidase assay results show that H₂S attenuates hypoxia-induced inhibition of neuronal NADPH oxidase expression (Fig. 5D). When the NADPH oxidase inhibitor DPI is applied, H₂S-mediated reduction of cellular ROS levels via Rac1 activation is abrogated (Fig. 5E). Considering that sulfhydration is a crucial pathway through which H₂S exerts its biological effects, we assessed the sulfhydration status of purified Rac1 protein. The results indicated that incubation with an H₂S donor markedly enhanced the sulfhydration of Rac1 protein (Fig. 5F). Furthermore, when neurons subjected to hypoxic conditions were pretreated with DTT, a sulfhydration inhibitor, the H₂S-induced suppression of NLPR3, GSDMD, and Caspase-1 expression was almost completely reversed (Fig. 5G–J). Fluorescence staining demonstrated that neurons under hypoxic stimulation generated increased levels of ROS, which were significantly diminished by H₂S treatment; however, this inhibitory effect was nullified by the addition of DTT (Fig. 5K, L). In summary, these findings suggest that H₂S inhibits Rac1 activity through sulfhydration modification.

### H₂S persulfidates Cys178 of Rac1 to attenuate inflammasome NLRP3 activation

**Fig. 6:**
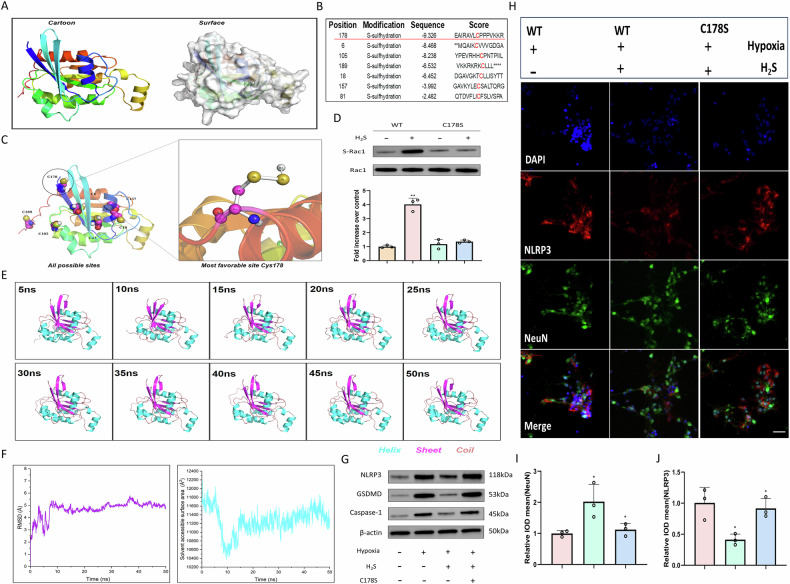
H₂S persulfidates Cys178 of Rac1 to attenuate inflammasome NLRP3 activation. **A**, **B** Structural prediction of Rac1 and lists potential sulfhydrylation sites with modification scores. **C** Molecular docking model identifying Cys178 as the most favorable persulfidation site. **D** Western blot and quantification of Rac1 persulfidation (S-Rac1) in wild-type (WT) and C178S mutant neurons. *n* = 3, *P < 0.05, **P < 0.01. **E** Kinetic simulation (50 ns) of Rac1 with H_2_S. **F** Analysis of radius of gyration and solvent accessible surface area, indicating structural activity alterations. **G** Western blot showing that H₂S fails to downregulate NLRP3, GSDMD, and Caspase-1 in C178S mutant neurons under hypoxia. *n* = 3, *P < 0.05, **P < 0.01. **H**–**J** Immunofluorescence and quantification of NLRP3 with NeuN in neurons of each group. Scale bar, 50 μm. *n* = 3, *P < 0.05, **P < 0.01.

To accurately determine the site of H₂S-induced sulfhydrylation on Rac1, we utilized the Uniprot and AlphaFold databases to predict the relevant cysteine residues. Our molecular docking model identified Cys178 as a primary site for persulfidation, evidenced by a docking score of −9.326 kcal/mol (Fig. 6A–C). Site-directed mutagenesis experiments demonstrated that H₂S pretreatment enhanced Rac1 persulfidation in neurons expressing wild-type Rac1, whereas this effect was abolished in neurons expressing the Rac1 C178S mutant (Fig. 6D). A 50 ns kinetic simulation with GYY4137 revealed subtle conformational changes in Rac1, along with significant alterations in the radius of gyration and solvent-accessible surface area, indicating potential changes in activity (Fig. 6E, F). Furthermore, GYY4137 failed to downregulate the levels of NLPR3, GSDMD, and Caspase-1 in neurons expressing the C178S mutant (Fig. 6G). Immunofluorescence colocalization analysis showed that the regulatory effect of H₂S on NLRP3 was significantly diminished following the C178S mutation (Fig. 6H–J). In summary, H₂S mitigates hypoxia-induced pyroptosis through the persulfidation of Cys178 on Rac1.

## Discussion

This study elucidates the pivotal role of H₂S in mitigating spinal cord pyroptosis subsequent to lumbosacral plexus nerve injury, revealing a novel mechanism whereby H₂S-mediated persulfidation of Rac1 reduces NLRP3 inflammasome activation. Through the integration of in vivo, in vitro, and molecular modeling methodologies, our findings identify the H₂S-Rac1 axis as a crucial redox-sensitive pathway regulating pyroptotic neuronal death. This research provides mechanistic insights into post-traumatic neurodegeneration and identifies potential therapeutic targets.

The substantial secondary loss of spinal neurons, with a mere 10–20% neuronal survival rate observed 12 weeks post-injury, underscores the critical need to elucidate the mechanisms underlying post-traumatic cell death [26, 27]. Pyroptosis, a lytic form of programmed cell death, has been identified as a significant pathological contributor in various neurological disorders, including spinal cord injury and peripheral neuropathy [28–30]. Our study reveals that LSPI induces progressive pyroptosis in spinal neurons, characterized by the upregulation of NLRP3, caspase-1, and GSDMD, in conjunction with neuronal loss. This finding is consistent with previous studies that associate NLRP3 inflammasome activation with secondary injury in spinal cord injury and ischemic brain injury, thereby indicating a conserved pathological mechanism across traumatic nerve injuries [31]. Notably, our in vitro hypoxia model replicated these molecular and morphological alterations, thereby validating pyroptosis as a hypoxia sensitive process in spinal neurons. The hypoxia-induced pyroptosis model further corroborates that mitochondrial ROS overproduction acts as a crucial priming signal for NLRP3 activation in neurons, aligning with the redox sensitive regulation of the inflammasome in ischemic brain injury [32, 33].

Hydrogen sulfide (H₂S), a recently identified gaseous signaling molecule within the body, plays a critical role in the regulation of various organ and tissue functions and metabolism. It exerts a wide range of biological effects by influencing cellular processes such as proliferation, apoptosis, and the inflammatory response [34–37]. Prior research has demonstrated that H₂S, at physiological concentrations, can mitigate inflammation in central nervous tissue and provide neuroprotection by inhibiting the production and release of pro-inflammatory cytokines and chemokines, including TNF-α and IL-1β [38]. In models of traumatic brain injury, H₂S has been shown to suppress the release of pro-inflammatory factors from astrocytes and microglia, enhance the production of anti-inflammatory cytokines such as IL-4 and IL-10, and potentially reduce neural tissue inflammation by modulating multiple signaling pathways [39]. In this study, we demonstrate that the biosynthesis of endogenous H₂S, mediated by CSE and 3MST, is downregulated in the spinal cord following LSPI, indicating a deficiency in protective sulfur-based signaling pathways. Supplementation with exogenous H₂S was found to attenuate pyroptotic markers, including NLRP3, caspase-1, and GSDMD, while preserving neuronal integrity in both in vivo and in vitro models. This effect is distinct from the direct antioxidant activity of H₂S, as our mechanistic studies reveal a post-translational regulatory role: H₂S induces the persulfidation of cysteine residues, a modification that dynamically alters protein function. Notably, the H₂S-mediated suppression of NLRP3 inflammasome assembly was negated by the persulfidation inhibitor dithiothreitol, thereby confirming the necessity of sulfur-based redox modifications in its anti-pyroptotic action.

A pivotal mechanistic discovery is the identification of Rac1, a member of the Rho-family GTPases, as a direct target of H₂S mediated persulfidation. The activation of Rac1 in its GTP-bound state facilitates the production of ROS through NADPH oxidase, serving as a critical upstream event in the priming of the NLRP3 inflammasome [40–43]. Inhibition of Rac1 activity via sulfhydration by H₂S may interfere with the upstream signaling pathways that culminate in the activation of the NLRP3 inflammasome. This hypothesis is corroborated by our findings, which demonstrate that H₂S treatment diminishes Rac1’s ability to bind GTP, while the sulfhydration inhibitor DTT negates the suppressive effect of H₂S on NLRP3 expression. Furthermore, site-directed mutagenesis and molecular dynamics simulations have elucidated that cysteine residue Cys178 is critical for the H₂S-induced conformational changes in Rac1, thereby disrupting its interaction with NLRP3. This cysteine-specific regulatory mechanism is consistent with emerging paradigms of redox-sensitive protein modulation [44], as illustrated by the H₂S-mediated persulfidation of p66Shc at Cys59 for oxidative stress mitigation [45] and PPARγ at Cys139 for metabolic regulation [46]. Our work extends this paradigm to pyroptosis control, positioning Rac1 persulfidation as a druggable node for neuroinflammatory disorders.

The H₂S-Rac1 axis emerges as a promising therapeutic target for neuroprotection in LSPI and other trauma-related neurodegenerative disorders. By focusing on redox-sensitive post-translational modifications, interventions based on H_2_S may interrupt the detrimental cycle of ROS-driven inflammation and pyroptosis, thereby potentially preserving neuronal viability and enhancing functional outcomes [47]. A significant advantage of persulfidation is its reversibility, which offers superior temporal and spatial control compared to genetic or irreversible pharmacological strategies [48, 49]. It is important to note that this study was conducted using a rodent model of LSPI, and translation to human applications necessitates validation in larger animal models, as well as an investigation into the pharmacokinetics of H₂S, including optimal dosing and delivery methods. Furthermore, although Rac1 is identified as a key mediator, the comprehensive profile of persulfidated proteins in the injured spinal cord, including potential interactions with other GTPases, remains unexplored. Future research should examine whether H₂S influences additional pyroptotic pathways or apoptotic mechanisms, given the critical nature of crosstalk between cell death pathways in trauma contexts.

In summary, our work uncovers a novel mechanism whereby H₂S inhibits spinal cord pyroptosis through Rac1 persulfidation at Cys178, disrupting NLRP3 inflammasome activation. This finding advances our understanding of redox-sensitive post-translational regulation in neuroinflammation and establishes the H₂S-Rac1 axis as a therapeutic node for preventing secondary neuronal loss after traumatic nerve injury.

## Supplementary information

WB-Supplemental

## Data Availability

The datasets generated and/or analyzed during the current study are available from the corresponding author on reasonable request.
