# Supplementary figures and images for: Hydrogen sulfide protects against spinal cord pyroptosis via persulfidation of Rac1 after lumbosacral plexus nerve injury
Source: Cell Death Discov. 2025 Oct 6;11:436. doi: 10.1038/s41420-025-02736-x (PMC12500895; doi:10.1038/s41420-025-02736-x)

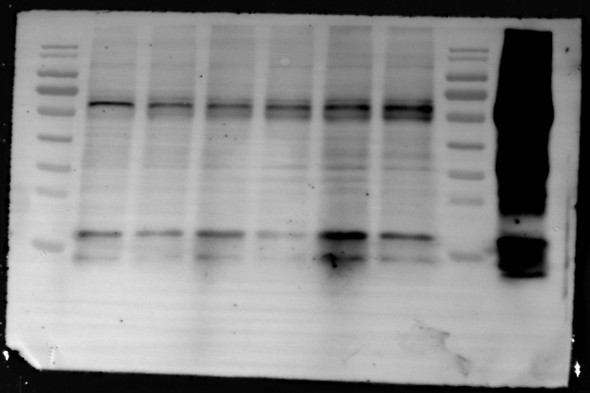


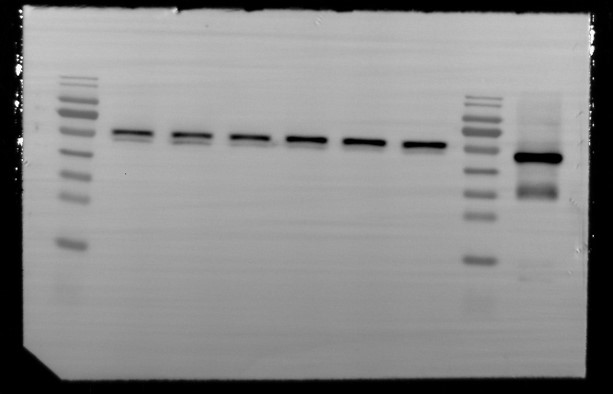

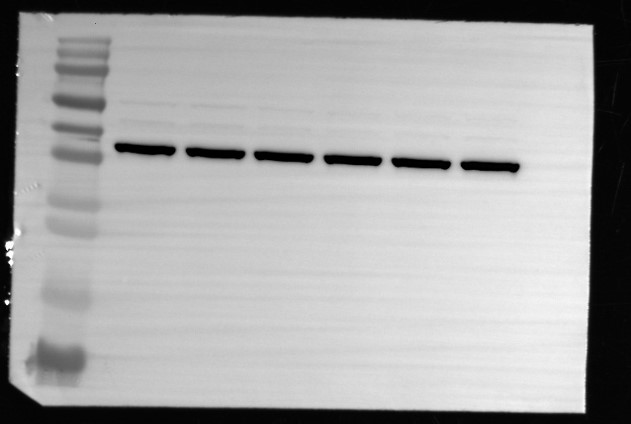

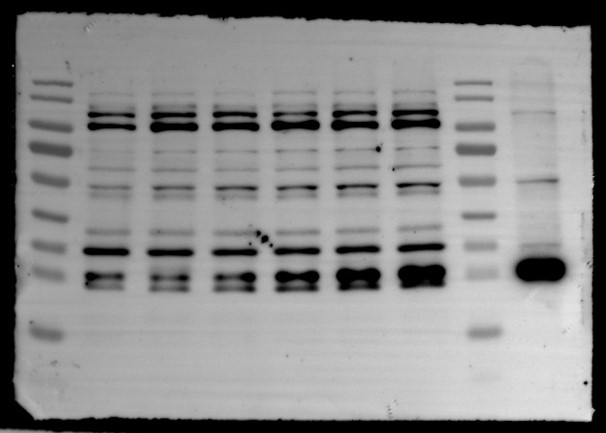


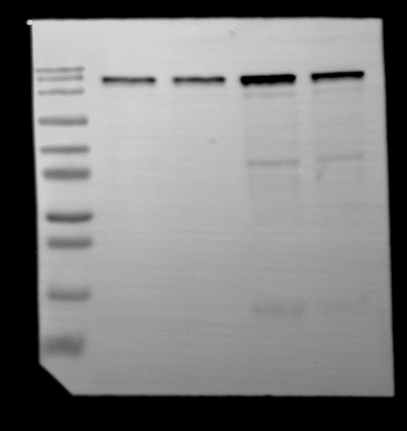

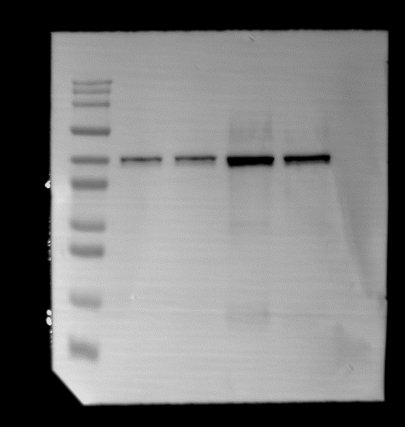

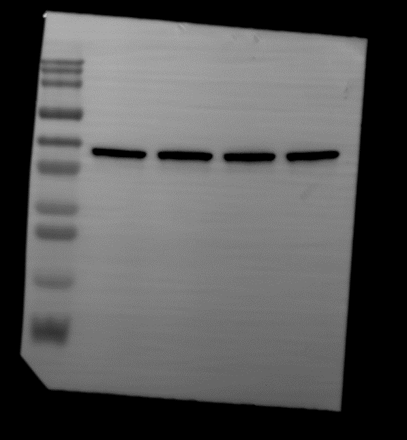

Supplement: Supplementary file 1 — WB-Supplemental [file 41420_2025_2736_MOESM1_ESM.docx]
